# Comparison of postoperative outcomes between different dissection techniques during laparoscopic cholecystectomy in rabbits: randomized study

**DOI:** 10.1590/acb396324

**Published:** 2024-10-25

**Authors:** María Camila Maldonado Vera, Monica Carolina Nery Wittmaack, Maria Eduarda Bastos Andrade Moutinho Conceição, Rachel Inamassu Faccini, Guilherme Sembenelli, Gabriel Luiz Montanhim, Mareliza Possa de Menezes, Cléber Kazuo Ido, Luiz Paulo Nogueira Aires, Gabriel João Unger Carra, Paola Castro Moraes

**Affiliations:** 1 Universidade Estadual Paulista – School of Agricultural and Veterinarian Sciences – Department of Clinic and Veterinary Surgery – Jaboticabal (SP) – Brazil.

**Keywords:** Adhesions, Cholecystectomy, Electrosurgery, Laparoscopy, Rabbits

## Abstract

**Purpose:**

Laparoscopic cholecystectomy (LC) is the gold standard for the treatment of gallbladder (GB) disease in small animals. The aims of this study were to investigate and compare the effect of different types of dissectors during LC in rabbits; electrothermal bipolar vessel sealing device (EBVS-LigaSure) and standard electrosurgical dissection (bipolar Maryland) for dissection of the GB in LC, correlating liver function tests (LFTs) in pre and postoperative periods (days 0, 3, 7, 15); macroscopic checking 15 days after surgery through necropsy; histopathological, bacteriological through bacterial growth by culture and intraoperative complications.

**Methods:**

Twenty rabbits were used, group (n = 10) using EBVS for GB dissection and cystic duct seal (GLL), and group (n = 10) using bipolar dissecting forceps and EVBS for cystic duct seal (GLE).

**Results:**

A higher concentration of alkaline phosphatase was observed on GLL 15 days after surgery when compared to GLE. In addition, GLE resulted in a higher concentration of alanine aminotransferase at three days when compared to GLL.

**Conclusion:**

In LC no significant statistical differences were found between EBVS and bipolar Maryland; both devices are equally safe and effective in LC. Further studies are required to evaluate the effectiveness of these devices in animals with gallbladder pathologies. Therefore, clinical studies are necessary.

## Introduction

The exponential growth of knowledge, skill and technology has completely changed the clinical practice of laparoscopic cholecystectomy (LC). LC techniques are approached to make the laparoscopic procedure safer and less technically demanding. Additionally, minimizing tissue injury during abdominal surgery has the benefit of reducing postoperative inflammatory response, pain, and adhesion formation^1^^,^ 2. The recent development of laparoscopic surgical techniques in different specialties has brought new concepts and alternatives to tissue dissection and hemostasis, allowing the evaluation and introduction of new laparoscopic devices using different energy modes. Electrothermal bipolar vessel sealing device (EBVS-Ligasure) represent a recent alternative system used in vessel sealing and closure systems in small animals^3^.

The traditional energy form used during laparoscopic dissection is electrocoagulation, applied through two systems. One of them is the bipolar system, which has some disadvantages, such as the unreliability of vessel sealing, difficulty in performing adequate dissection with the bipolar forceps, and tissues tend to adhere to the blades; most instruments have a very limited ability to vaporize or cut tissue^4^. Because of its documented risks, especially liver injury, damage to adjacent organs such as the common bile duct, stomach and intestines, as well as association with smoke, charring and biological hazards, there is a growing interest in finding surgical alternatives^5^. In that context, the EBVS was designed as a safe alternative to the traditional electrocautery dissection^2^.

To the best of our knowledge, no study has been conducted to evaluate the effect of different types of dissectors used in LC in veterinary medicine, evaluating and correlating the total surgery time, liver alterations of macroscopic, histopathological, bacteriological, and changes in liver function tests (LFTs). Therefore, the objective of this study was to compare the possible aspects of the effect, efficiency, safety, and advantages of different types of dissectors (EBVS-Ligasure *vs*. standard bipolar electrocautery forceps–Maryland) for dissection of the gallbladder from the hepatic fossa in LC.

## Methods

Twenty adult New Zealand white male rabbits, weighing between 3 and 4 kg, 2 years old, were used. The project was approved by the Ethics Committee on the Use of Animals of the Faculty of Agricultural and Veterinary Sciences of the Universidade Estadual Paulista “Júlio de Mesquita Filho”, Jaboticabal *Campus*, SP, Brazil, and carried out in accordance with standards required by the National Council for Animal Experimentation Control, protocol no. 016256/19.

These 20 patients were randomly assigned to two groups. The GLL group included 10 patients for gallbladder dissection with an EVBS, while the GLE group included 10 patients for gallbladder dissection with an electrosurgical device Maryland. Previously to the laparoscopy technique, blood samples were collected from the 20 rabbits for the evaluation of enzymatic and biochemical parameters indicating liver function for analysis of: total protein, albumin, gamma-glutamyl transferase (GGT), and alkalische phosphatase (ALP), alanine aminotransferase (ALT), aspartate aminotransferase (AST), total bilirubin and direct bilirubin, fibrinogen and blood count in the immediate postoperative period, on the third (T3), seventh (T7), and 15th day (T15) after the procedure.

In the perioperative period, postoperative complications, surgical time, time of dissection of the gallbladder of the hepatic fossa, and total time of surgery were evaluated. Fifteen days after the procedure, the rabbits were euthanized. During the necropsy, the abdominal cavity was evaluated macroscopically, samples were collected by rubbing a sterile cotton swab on the gallbladder bed resection for counting microorganisms, and, subsequently, histopathological evaluation was performed.

The choice of animal model for an experiment is determined by factors such as cost, technical feasibility of the procedure, scientific principles, available database and suitability of models for the project^6^. Rabbits are small Lagomorph mammals^7^. Among several strains, the New Zealand white strain (*Oryctolagus cuniculus*) is frequently used for research activities, as a model for in-vivo studies, as well as a suitable model for experimentation in surgery^8^^,^ 9, and for training in laparoscopic and open surgery^10^^–^ 12. These bloodlines are less aggressive in nature and have fewer health problems compared to other breeds; in addition to complying with anatomical characteristics comparable to dogs and cats weighing less than 5 kg or small or medium size, with ease of use and handling.

It is for these reasons that this work used rabbits as an experimental model, since the size of the abdominal cavity of these animals mimics the difficulty encountered in laparoscopic techniques for dogs and cats. In addition, LC in infants requires highly specialized skills and great experience. Furthermore, the extra difficulty due to the small space seems an additional advantage for surgeons to adapt to the very slow controlled movement of instruments necessary in endoscopic surgery^13^.

### Setting

Rabbits did not fast prior to the procedure due to the rare incidence of emesis^14^^,^ 15. As pre-anesthetic medication, morphine 1 mg/kg and acepromazine 0.05 mg/kg, via intramuscular (IM), were administrated. After 20 minutes, anesthetic induction and maintenance were performed with isoflurane and maintained under spontaneous ventilation. Tramadol hydrochloride 4 mg/kg, meloxicam 0.1 mg/kg, and enrofloxacin 5 mg/kg were administered subcutaneously; for the postoperative period, tramadol hydrochloride 4 mg/kg was administrated subcutaneously every 8 hours. For three days, meloxicam 0.1 mg/kg was administrated once a day subcutaneously for two days, and enrofloxacin 5 mg/kg was administrated every 12 hours subcutaneously for seven days.

**Figure 1:**
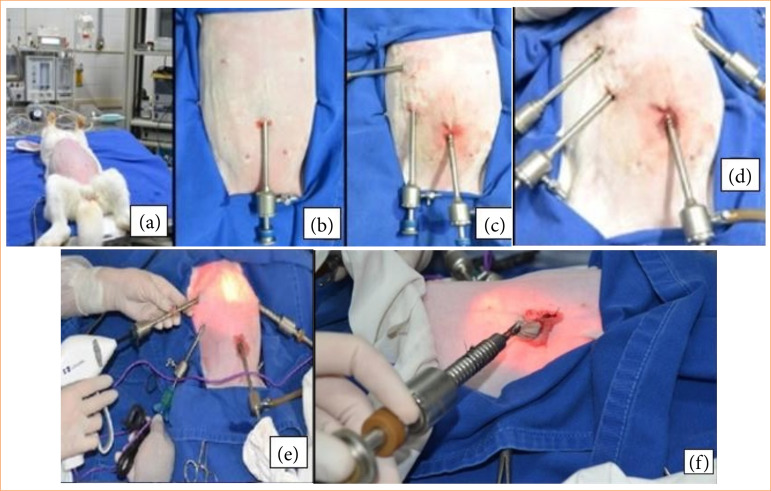
Laparoscopic cholecystectomy in a rabbit. **(a)** Patient in reverse Trendelenburg position. **(b)** Access site of the first portal below the umbilical scar. **(c)** Access location of the first and second portal. Insufflator, optics and light source coupled to the first port cannula. **(d)** First, second, third and fourth port access location. **(e)** Insertion location of the four access ports and atraumatic forceps. **(f)** Removal of the gallbladder inside the recovery bag by opening the 10mm.

Source: Elaborated by the authors.

After anesthetic induction, the rabbits were placed in dorsal recumbency, and hair from the ventral and lateral portion of the abdomen was clipped; the patients were placed in reverse Trendelenburg position with the right side up^3^^,^ 14 ^,^ 15 to allow the stomach and small intestine to move caudally. Previous and definitive antisepsis was performed with 2% chlorhexidine and 70% alcohol, followed by placement of surgical drapes. A skin incision of 0.5 cm was made in the ventral abdominal midline, 1 cm caudal to the umbilical scar. After subcutaneous dissection, a small 3–4 mm incision was made in the linea alba, penetrating the abdominal cavity. Then, a 5-mm trocar was introduced through, in which the 5-mm laparoscope was placed, coupled with the microcamera and the light source (Fig. 1). The insufflator was attached to the cannula, inflating the peritoneal cavity with carbon dioxide (CO_2_) at a speed of 2 L/min, maintaining abdominal pressure between 5 and 10 mmHg. Initially, a limited exploration of the abdomen was carried out with special attention to the liver, gallbladder and extrahepatic biliary tree. Through the optics, the insertion of other access portals was also observed.

Two 5-mm access portals were inserted into the right abdominal cranial quadrant, located 3 and 5 cm lateral to the midline and 3 and 4 cm cranial to the umbilical scar, positioned to obtain a triangulation around the anticipated location of the gallbladder. Through these two portals, Babcock atraumatic grasping forceps were inserted for elevation of the hepatic lobe and subsequent visualization of the hepatic fossa and for manipulation of the gallbladder.

A fourth 5-mm port was inserted into the left abdominal quadrant (near the costal arch, 5 cm lateral and 5 cm cranial to the umbilical scar). This port was used to introduce standard electrosurgical dissection (bipolar Maryland) and EBVS-LigaSure for diffusion around the cystic duct and subsequent divulsion of the gallbladder, insertion of the EBVS for occlusion of the cystic duct and, at the end, the recovery bag for removal of the gallbladder.

After identification of the gallbladder and cystic duct, two different techniques for dissection of the gallbladder and two different methods for occlusion of the cystic duct were used.

For all techniques, the gallbladder was grasped in the same way, first using an atraumatic forceps to grasp and retract the gallbladder fundus, in the cranial and right lateral direction, over the liver dome. The infundibulum was identified, seized, and later retracted laterally towards the lower right quadrant using another atraumatic forceps. This maneuver exposed the Calot triangle. The peritoneum covering the infundibulum of the gallbladder was incised and released by the Maryland forceps in both groups. The triangle was dissected to expose the cystic duct, cystic artery, and lymph node. Once these structures were carefully identified, the cystic artery was sealed with EBVS in both groups, then continued with dissection of the triangle until it was determined that the only remaining structure connected to the gallbladder was the cystic duct.

**Figure 2:**
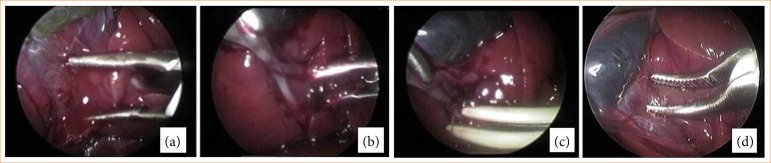
Laparoscopic cholecystectomy technique in the GLE group. **(a)** Using the Maryland bipolar dissection forceps to initiate the dissection through the Calot triangle, **(b)** the cystic duct occlusion was performed with electrothermal bipolar vessel sealing device, with two occlusions around the duct. **(c)** The cystic duct transection with the electrothermal bipolar vessel sealing device. **(d)** The peritoneal attachments between the gallbladder and its liver bed were dissected using the Maryland bipolar forceps in the direction of the triangle of Calot to the bottom of the gallbladder.

Source: Elaborated by the authors.

**Figure 3:**
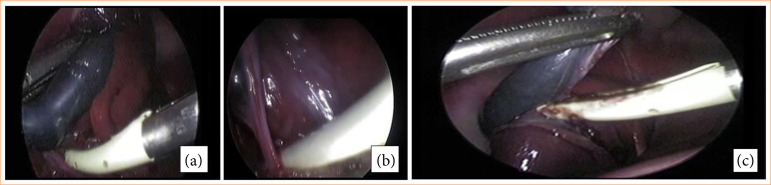
Laparoscopic cholecystectomy technique in the GLL group. **(a)** Dissection through the triangle of Calot. **(b)** Occlusion and transection of the cystic duct. **(c)** The peritoneal attachments between the gallbladder and its liver bed were performed with a electrothermal bipolar vessel sealing device.

Source: Elaborated by the authors.

The GLE group (n = 10) was submitted to the CL technique using the standard electrosurgical dissection (bipolar Maryland) to initiate the dissection through the Calot triangle, the cystic duct occlusion was performed with a EBVS-LigaSure. Two occlusions were performed around the duct. The cystic duct transection was performed with the EBVS-LigaSure forceps cutting function in the occlusion near the gallbladder, leaving two one distal occlusions in the cystic duct. Then, after occlusion of the cystic duct, the peritoneal attachments between the gallbladder and its liver bed were dissected using the bipolar Maryland forceps in the direction of the triangle of Calot to the bottom of the gallbladder (Fig. 2). For the second technique proposed, in the GLL group (n = 10), dissection through the triangle of Calot and occlusion and transection of the cystic duct were performed with a EBVS-LigaSure (Fig. 3).

**Figure 4:**
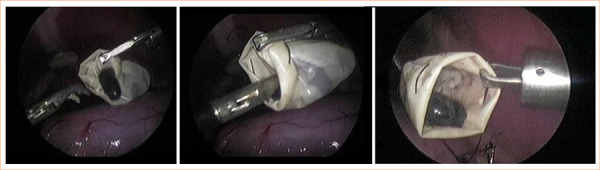
Placement of gallbladder within recovery bag after complete dissection and section of the cystic duct.

Source: Elaborated by the authors.

After complete divulsion of the gallbladder and sectioning of the cystic duct, it was placed inside the recovery bag (Fig. 4). The recovery bag was removed by enlarging the right portal incision.

The portals on the right and left side were then removed. Before endoscopic removal, the hepatic fossa was examined to verify the absence of hemorrhage and bile leakage. The deflation of the abdominal cavity was carried out. The cannula and endoscope were removed. The abdominal musculature was sutured by a “Sultan” suture with 2-0 poliglecaprone thread and the skin by a simple interrupted suture with 2-0 nylon.

### Intraoperative complications

Intraoperative complications including abundant bleeding during dissection and obscuring the surgical area, impeding further dissection. Bleeding was managed by electrocoagulation until the bleeding stopped, and the surface of the liver bed was washed with saline. Other intraoperative complications included biliary leak, gallbladder perforated, lesions of the bile ducts combined with major vascular damage bile, laceration of the liver bed during LC.

### Postoperative assessment

The animals underwent abdominal ultrasound and blood sampling taken from the jugular vein preoperatively day 0, and in the immediate post-operative period 3, 7 and 15 days after the operation, evaluating in ultrasound procedure-related postoperative complications, which were classified as hemorrhage, biliary leak, intra-abdominal abscess, or bile peritonitis; in the evaluating of blood the enzyme level alterations were compared: alkaline phosphatase (ALP), ALT, AST, GGT, direct bilirubin, and total bilirubin.

### Necropsy analyses

Fifteen days after the procedure, the rabbits were euthanized, by catheterizing the right atrial vein1 for the administration of propofol^2^ in order to achieve a deep anesthetic plane verified by apnea and total mydriasis. Then, by the same intravenous route, potassium chloride^3^ was administered until permanent cardiorespiratory arrest. Then, necropsy was carried out through a midline abdominal incision. The area of adhesion was expressed on a scale ranging from 0 to 4 (Table 1).

**Table 1:** Adhesion scoring system.

Grade	Description of grade
0	No adhesions, no omentum adhesions (neither to the abdominal wall nor to the liver bed)
1	Thin or narrow, easily separable adhesions
2	Thick adhesions, limited to liver bed
3	Thick and widespread adhesions (omentum to liver bed, trocar site or abdominal wall)
4	Thick and widespread adhesions plus adhesions of viscera to the liver bed anterior or posterior abdominal wall (or both) located

Source: Elaborated by the authors.

Both the professionals who performed the necropsy, as well as the pathologists, were blinded to type of energy source used in every surgical procedure in the different animals.

### Histologic findings

Each liver sample from 20 animals (containing all the hepatic lobes) were submitted to histologic examination (Table 2). The sample from the entire liver was removed and immersed in a 3% paraformaldehyde solution, in 0.1M phosphate buffer, and fixed by immersion for three days. The samples Cateter BD Angiocath^®^ 22 G (Becton, Dickinson Indústria Cirúrgica LTDA, Juiz de Fora, MG, Brazil); Propovan (Cristália Produtos Químicos e Farmacêuticos LTDA); and potassium chloride (Darrow Lab. S/A, Botafogo, RJ, Brazil) were dehydrated in increasing alcohol solutions (50 to 100%) cleared in xylene and embedded in paraffin and then cut coronally in a rotating microtome in 5-µm thick sections. The sections were extended on histological slides.

**Table 2:** The parameters evaluated in histological examination.

Score	giant cells	necrosis	fibrosis
1	None	None	No fibrosis
2	Difficult to find	Mild	Minimal, loose fibrosis
3	Easy to find	Moderate	Moderate fibrosis
4	Many	Intense or severe	Florid dense fibrosis

Source: Elaborated by the authors.

During the histologic examination, the presence or absence of siderophags was also evaluated.

### Bacteriological evaluation

#### Microbiological count of total aerobes

On the day of the necropsy, samples were collected by rubbing a sterile swab on the gallbladder bed resection. After collection, the samples were transported in a sterile tube containing 0.1% peptone solution to the Laboratory of Microbiology of the Department of Veterinary Pathology at Universidade Estadual Paulista “Júlio de Mesquita Filho”, Jaboticabal *campus*.

The microorganism count was performed by surface plating (“Spread Plate”). For this, initially a serial decimal dilution (1:10) was performed, in which 500 μL of the sample in the tube containing the swab and peptone water was transferred to another tube containing 4.5 mL of 0.1% peptone water, and so on, successively. This dilution is necessary to plate with countable colony forming units (CFU), as established by APHA16, which recommends up to 2 CFU/cm2. After the dilutions, 100 μL of each previously diluted tube was inoculated into Petri dishes containing “brain heart infusion” culture medium. Then, the samples were spread and homogenized in the plates containing the culture media using a Drigalski loop.

### Statistical analysis

Statistical analyses were performed using Software R (version 3.6.3).

In the blood chemistry and blood count data, the effect of the surgical technique and the day was evaluated by the Friedman’s test, and their interaction by the Kruskal-Wallis’ test and Dunn’s post-test. When significant, the interaction is presented in figures made with the help of the GraphPad Prism statistical software (version 6.01).

Data on surgical times, adherence score, and hepatic lobe after gallbladder dissection at necropsy, and giant cell score, necrosis and fibrosis on histopathology were evaluated between groups using the Mann–Whitney’s test. The proportion of intraoperative complications, culture and complication observed by ultrasound and siderophagus in the histopathology were compared between groups by Fisher’s exact test.

The resulting values for each variable are presented as the median ± the interquartile range (IQR). In all tests, significance was set at *p* ≤ 0.05.

## Results

**Figure 5:**
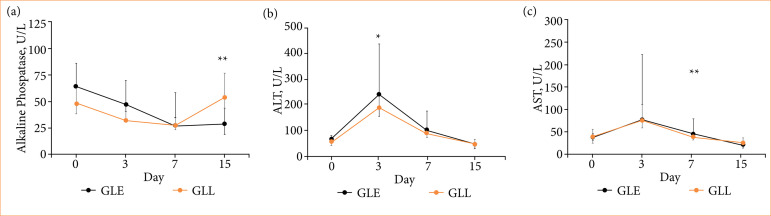
Median interquartile range of **(a)** alkaline phosphatase, **(b)** alanine aminotransferase (ALT), and **(c)** aspartate aminotransferase (AST), as a function of days after gallbladder dissection surgery using the LigaSure + electrosurgical (GLE) or LigaSure + LigaSure (GLL) technique in rabbits.

No postoperative morbidity or mortality occurred in any of the patients studied. All patients were hemodynamically stable during the operations, and none of them needed other medication than the planned protocol. No rabbits required surgical revision due to hepatic duct leakage, bleeding complication or bile peritonitis postoperative. Necropsy showed no peritonitis, or extra-hepatic biliary tract rupture. The techniques LigaSure + Electrosurgical (GLE) and LigaSure + LigaSure (GLL) showed no differences in surgical times, findings at necropsy, percentage of intraoperative complications, percentage of bacterial growth by culture. In both techniques, there was growth of bacteria in two of the 10 rabbits, and % of complications were detected by ultrasound (*p* > 0.05; Table 3). Values for GGT, AST, total bilirubin, direct bilirubin, and fibrinogen were similar for both techniques on all evaluation days (*p* > 0.05; Table 4; Fig. 5). However, in the GLL group, an increase in the concentration of ALP was observed 15 days after surgery (53.50 ± 15.75 vs. 29.00 ± 41.25 U/L) (*p* = 0.004; Fig. 5a), when compared to GLE, however, according to normal parameters in rabbits. On the other hand, GLE resulted in a higher concentration of ALT at three days (243.50 ± 193.25 U/L) when compared to GLL (190.00 ± 34.00 U/L) (*p* < 0.001; Fig. 5b). The GLE and GLL techniques did not show differences in the score of giant cells, necrosis and fibrosis during histopathology, and similar percentage of presence of siderophores (*p* > 0,05; Table 5).

**Table 3:** Median ± interquartile range of surgical times, and scores of necropsy findings and complications in rabbits after gallbladder dissection by two surgical techniques.

technique	GLE	GLL	*p*-value
Surgical times (minutes)			
Dissection-Cholecistectomy	11.00 ± 6.00	11.50 ± 7.00	0.675
Total time	33.00 ± 19.25	33.50 ± 28.50	0.820
Necropsy			
Adhesions	1.00 ± 3.25	1.50 ± 3.75	0.782
Intraoperative complication (%)	40.00 (4/10)	20.00 (2/10)	0.628
Culture (%)	10.00 (2/10)	20.00 (2/10)	0.990
Ultrasound complication (%)	20.00 (2/10)	20.00 (2/10)	0.999

GLE: LigaSure + electrosurgical; GLL: LigaSure + LigaSure. Source: Elaborated by the authors.

**Table 4:** Median ± interquartile range of blood chemistry in rabbits submitted to two surgical techniques.

Technique	GLE	GLL	*p*-value		T × D
			Technique	Day	
GGT (U/L)	1.00 ± 0.85	1.00 ± 0.00	0.317	0.144	0.055
Alkaline phosphatase (U/L)	40.50 ± 44.25	39.00 ± 21.25	0.318	0.241	0.004
ALT (U/L)	82.00 ± 104.50	77.50 ± 65.75	0.323	0.111	< 0.001
AST (U/L)	44.00 ± 47.50	37.50 ± 38.75	0.999	0.112	0.059
Total bilirubin (mg/dL)	0.04 ±0.04	0.06 ±0.07	0.318	0.145	0.236
Direct bilirubin (mg/dL)	0.02 ± 0.01	0.02 ± 0.02	0.317	0.308	0.255
Fibrinogen (mg/dL)	0.40 ± 0.40	0.40 ± 0.40	0.328	0.120	0.359

T × D: interaction between the type of technique and the days of assessment evaluated by the Kruskal-Wallis’ test; GLE: LigaSure + electrosurgical; GLL: LigaSure + LigaSure; GGT: gamma-glutamyl transferase; ALT: alanine aminotransferase; AST: aspartate aminotransferase. Source: elaborated by the authors.

*p < 0.001, **p = 0.004 obtained by the Dunn’s test. Source: Elaborated by the authors.

**Table 5:** Median ± interquartile range of giant cell score, necrosis and fibrosis in the histopathological exam and the presence of siderophage in rabbits after vesicle dissection by two surgical techniques.

Technique	GLE	GLL	*p*-value
Giant cells	3.00 ± 0,75	3.00 ± 1.00	0.312
Necrosis	3.00 ± 0.00	3.00 ± 1.00	0.167
Fibrosis	2.00 ± 0.00	2.00 ± 0.00	0.995
Siderophage (%)	10.00 (1/10)	20.00 (2/10)	0.999

GLE: LigaSure + electrosurgical; GLL: LigaSure + LigaSure. Source: Elaborated by the authors.

## Discussion

LC, as any surgical procedure, is not 100% safe nor free from difficulties. Among the serious complications related to this procedure, there are retained stones in common bile duct and duct injuries, which are not easy to recognize during surgery and are usually detected post-surgery^17^. LFTs are generally used postoperatively as an indicator of liver function and of duct obstructions and iatrogenic injuries. The sensitivity of this tests above all ALP in predicting biliary obstruction has been shown to be high^18^. However, in earlier studies, an alteration in liver function tests of up to 70% has been reported with no adverse clinical outcome^19^. Gallbladder surgery is not the only procedure associated with elevated postoperative LFTs. Other abdominal laparoscopic procedures have been also associated with these alterations^20^.

Elevation of the liver enzymes represents hepatocellular injury. According to several studies, this elevation may be related to increased pneumoperitoneum pressure by carbon dioxide formed during the surgery, the squeezing effect on the liver by cranial retraction of gallbladder during LC, excessive diathermy (heat energy) used during the procedure (cauterization of the liver bed for hemostasis), manipulation of external bile ducts and effects of general anesthesia (which induced hepatic hypoperfusion)^21^^,^ 22.

Our study demonstrated that transient elevation of LFTs could occur after LC, however there are no significant differences between the dissectors compared. These results agree with the study by Mazahreh et al. made in people^23^. It found no statistical differences between the different types of dissectors in the alterations of ALT and AST after LC. Nevertheless, the alteration in LFTs could be contributed to using electrocautery device, as in our case, in which an increase in ALT is evidenced on the third postoperative day only in the dissection group with Maryland Bipolar electrocautery. Hochstadetr et al.^24^ demonstrated a significant rise in liver enzymes (ALT) after surgery, in both electrocauteries as ultrasonic cutter. However, postoperative values of these two enzymes were significantly higher in patients operated on using the electrocautery. Similarly, Al-Abbadi et al.^22^ showed a significant elevation in LFTs post-LC in goats. According to the authors, one reason for the increase was the use of diathermy for hemostatic control.

In our results, the elevation of ALT on the third day after surgery by GLE returned to normal levels on day 15, as in the study by Mazareh et al.^23^, in which all patients returned to normal values of LFTs after one week postoperatively. However, the elevations in our study for the GLL of alkaline phosphatase were evidenced from postoperative day 15 (but still within the reference interval of biochemical values in New Zealand rabbit serum)^25^^,^ 26. Studies such as that of Ahmad et al.^27^ identified that liver enzyme values returned to normal after three weeks, Al-Jaberi et al.^28^, and Tan et al.^29^ at 72 hours, Maleknia and Ebrahimi^30^ performed measurements up to 48 hours after surgery and determined that they still did not return to preoperative levels, and Milheiro et al.^31^ showed normalization in levels up to 30 days after surgery. Therefore, this evidence shows the need to evaluate the levels of LFTs for a longer period. Our result shows that a longer follow-up period is needed to conclude a biliary tract injury; the measurement of elevated postoperative enzymes after three or 15 days does not necessarily indicate a complication or haste in unnecessary interventions.

The rate of electrosurgical complications during delivery of energy to the surgical site is estimated to be 25.6% and is the second most common laparoscopic complication after a misplacement of trocar or Veress needle, which is 41.8%^32^. Injuries during laparoscopic electrosurgical procedures are mechanical trauma, or electrothermal injuries^33^. After abdominal surgery, adhesion formation seems to be one of the major pathologic changes that patients suffer^34^. Diamantis et al.^35^ compared the results of thermal injury by monopolar electrocoagulation, bipolar electrocoagulation, ultrasonic scalpel and LigaSure vessel sealing system (EBVS) in the gastric vessels of a rabbit model. They noticed that EVBS demonstrated the mildest side thermal injury and the fastest healing process on the neighboring gastric wall. These investigators also noted greater thermal injury and inflammatory response by ultrasonic scalpel than by EBVS on postoperative days 7 and 14. According to these data, EBVS causes less tissue damage than ultrasonic scalpel and electrocautery. Also, Landman et al.^36^ reported in a comparative study in a porcine model that the least acute collateral tissue injury was achieved by the EBVS when compared to bipolar electrosurgery devices.

In our study, there were no significant differences in the formation of adhesions, in the histologic markers of inflammation and fibrosis in the liver bed. This is also consistent with previous studies in which it was shown there was no difference in adhesion formation between CO_2_ laser and electromicrosurgery, according to Luciano et al.^37^, or between ultrasonic scalpel and steel scalpel according to Tulandi et al.^38^. In a study by Schemmel et al.^39^ in which they bought several laparoscopic surgery instruments in terms of tissue injury and adhesion formation in a rabbit model, it also found no differences in terms of hemostatic properties, coagulation necrosis, or adhesion formation. Vetere et al.^40^ performed a study in rabbits who underwent injuries by using ultrasonic energy on one uterine horn and the adjacent pelvic sidewall and using monopolar energy on the opposite side. They concluded that there was no significant difference found in the pathological adherence scores between the different energy sources.

Establishing hemostasis is critical to improving clinical outcomes and providing a clear field for adequate visualization. The ability to obtain hemostasis using EBVS has represented a great advance in the fields of both open and laparoscopic hepatobiliary surgery^41^. Our study suggests that performing a LC with EBVS is beneficial in reducing intra-operative bleeding. The improved hemostasis is likely due to EBVS vessel sealing properties, although our results showed no significant differences regarding intraoperative complications among GLE and GLL groups. In our study, none of the cystic ducts sealed with the EBVS leaked in any patient (GLE and GLL) during the evaluated time period, which is in accordance with studies using EVBS devices for cystic duct ligation in people that have reported successful results^42^^–^ 44, likewise in the study by Schulze et al.^44^ in which there was no biliary leakage after closing the cystic duct with EBVS and was associated with minimal complications. Turial et al.^43^ showed that closing the cystic duct using EBVS (LigaSure) is feasible and effective in LC in children. In the study by Marvel and Monnet^42^, the use of a vessel-sealing device on the ligation and transection of cystic ducts in healthy canine cadavers is comparable to 10 mm metallic surgical clips. Despite this, there are other studies, such as the one by Hope et al.^41^, and Matthews et al.^45^, in porcine using EBVS for cystic ligation and common bile duct have documented bile leakage.

All surgeries have an inherent risk of infection, but the risk is relatively small in case of laparoscopic or minimal access surgery^46^. In the present study, there were no statistical differences regarding the percentage of bacterial growth per culture, which can be explained, according to Targarona et al.^47^, who indicated that laparoscopic surgery is associated with better preservation of the immune system, a lower incidence of complications and a better postoperative outcome compared with conventional open surgery, consequently lowering the incidence of infectious complications. Even though carbon dioxide pneumoperitoneum influences the mechanisms of the peritoneal response, it does not appear to have a harmful effect in terms of intra-abdominal infection, and Castro et al.^48^ indicated that LC is associated with a lower incidence of infectious complications compared to open surgery.

Further, according to Alam et al.^49^, LC is associated with a low risk of infection, and the few that exist are most seen at the port site through which the gallbladder was extracted. With innovation of minimal invasive surgery, the risk of wound infection has decreased considerably. Most of the authors, like Mir et al.^50^, Shindholimeth et al.^51^, Sathesh-Kumar et al.^52^, Den Hoed et al.^53^, and Taj et al.^54^, reported that the incidence of port site infections is more than 5%, but some authors like Colizza et al.^55^ have reported that this incidence is less than 2%. In turn, Sharma et al.^46^ and Al-Naser^56^ indicate an incidence of port site infections of 4.5%.

However, it is necessary to consider the useful life of the surgical instrument, taking into account that it is being sterilized and reused in each surgery in humans. Abdelaziz et al.^57^ in a study carried out during total hip and knee arthroplasty determined that bacterial contamination of electrocautery tips is relatively high. However, in the future, more in-depth studies would be needed to determine whether bipolar electrocautery or EBVS generate repercussions in its use due to a certain number of sterilizations and reuses, but this was not the objective of the present study.

With the results of the present study, it is concluded that neither bipolar electrosurgery nor EBVS is superior to one another in terms of the incidence in surgical times, findings at necropsy, percentage of bacterial growth by culture, nor in the score of giant cells, necrosis and fibrosis during histopathology, and similar percentage of presence of siderophores.

Likewise, it showed that elevation of LFTs (ALP, ALT) can happen after LC, but it is transient and clinically silent in patients with normal liver function. It seems that the dissector type has no effect on the alteration of LFTs. These enzyme elevations seem to be attributable to other factors, mainly to the negative effects of the pneumoperitoneum on the hepatic blood flow. Although these changes do not seem to be clinically important, care should be taken before deciding to perform LC in patients with hepatic insufficiency. Overall, the procedure is safe without any morbidity and mortality rate; both devices are equally safe and effective in LC.

Despite not having significant differences between the dissectors, our results with no leakage suggest that the use of the EBVS is safe and effective for closure and division of the cystic duct in LC in rabbits. Also, our study suggests the EBVS decreases intraoperative bleeding compared to conventional bipolar dissector. Our data indicated that the complete LC procedure can be performed with the EVBS. The instrument is suitable for dissection, sealing, and division of the cystic duct and artery and the tissue anchoring the gallbladder to the gallbladder bed in the liver^2^.

## Conclusion

With the results of the present study, it is concluded that neither Bipolar bipolar electrosurgery nor EBVS is superior to one another in terms of the incidence in surgical times, findings at necropsy, percentage of bacterial growth by culture, nor in the score of giant cells, necrosis and fibrosis during histopathology, and similar percentage of presence of Siderophoressiderophores.

## Data Availability

The data are available from the corresponding author upon reasonable request.
